# Selective Advantages of Synapses in Evolution

**DOI:** 10.3389/fcell.2021.726563

**Published:** 2021-08-20

**Authors:** Leonid L. Moroz, Daria Y. Romanova

**Affiliations:** ^1^Departments of Neuroscience and McKnight Brain Institute, University of Florida, Gainesville, FL, United States; ^2^Whitney Laboratory for Marine Biosciences, University of Florida, St. Augustine, FL, United States; ^3^Lab of Cellular Neurobiology of Learning, Institute of Higher Nervous Activity and Neurophysiology of Russian Academy of Sciences, Moscow, Russia

**Keywords:** nervous system evolution, endoplasmic reticulium, neurotransmitters, immune synapse, ctenophores, placozoa, lipids, mitochondria-associated membranes

The common ancestor of all Metazoa (Urmetazoan) was a nerveless animal (Mackie, 1970, 1990; Moroz, 2009). This hypothetical Urmetazoan likely used electrical and chemical communications for behavioral control as in the present-day placozoans or sponges. In placozoans, non-synaptic signaling is mediated by small secretory peptides and low molecular weight transmitters, including NO, ATP, glutamate, glycine, GABA (Nikitin, 2015; Varoqueaux et al., 2018; Moroz et al., 2020a,b, 2021b; Romanova et al., 2020a,b).

Emerging evidence suggests that neurons evolved more than once from secretory cells (reviewed by Moroz, 2014, 2021). Such events might independently occur about 550–540 million years ago in ancestors of three basal metazoan lineages: ctenophores, cnidarians, and bilaterians (Moroz et al., 2021b). The separations of each of five basal metazoan lineages likely happened within a relatively short geological interval, perhaps, even <10 million years. Thus, the outcome of the highly debated topic—the identification of the sister lineage for all Metazoa (i.e., ctenophore-first or sponge-first hypotheses, see details in Whelan et al., 2015, 2017; Halanych et al., 2016; Telford et al., 2016; Kapli and Telford, 2020; Redmond and McLysaght, 2021)—does not challenge the fact of extensive parallel evolution of neural organization within the majority of animal phyla. It also does not challenge the hypothesis of the independent origins of neurons considering remarkably different molecular toolkits for neural cell types across basal metazoans (Moroz et al., 2014) and the broadening definitions of neural systems (Miguel-Tomé and Llinás, 2021).

The surprising corollary of the neural polygeny hypothesis is independent origins of synapses (Moroz and Kohn, 2016; Moroz et al., 2021b). But how had the synaptic organization from secretory cells happened in early animal evolution? What were the selective advantages of synaptic vs. paracrine secretory communications? Some particular “benefits” of synapses are apparent, and some are not. We think that the extension of the endoplasmatic reticulum and growing lipids' diversity in early secretory cells paved the way to versatile and divergent neuronal and synaptic evolution.

## Advantages of Synapses in Neural Evolution

*First*, both ***speed***and more localized, faster delivery of intercellular signals are probably among the most prominent selective advantages of synapses in evolution compared to volume transmission. Consequentially, selective “*benefits”* of shorter and anatomically restricted transmission enabled more precise control [and homeostasis] of transmitter concentrations. There is always a trade-off between the chemical stability vs. the rate of transmitter's chemical inactivation in given microenvironments [e.g., oxidation for monoamines (Burbulla et al., 2017; Riessland et al., 2017), hydrolysis of acetylcholine, or proteolysis for peptides].

*Second*, both the ***exocytosis*** and regulation of chemical transmission within small contained volumes would require fewer resources and might be energetically more favorable than producing and releasing a larger pool of transmitters into extracellular spaces to compensate for their diffusion.

**Figure 1:**
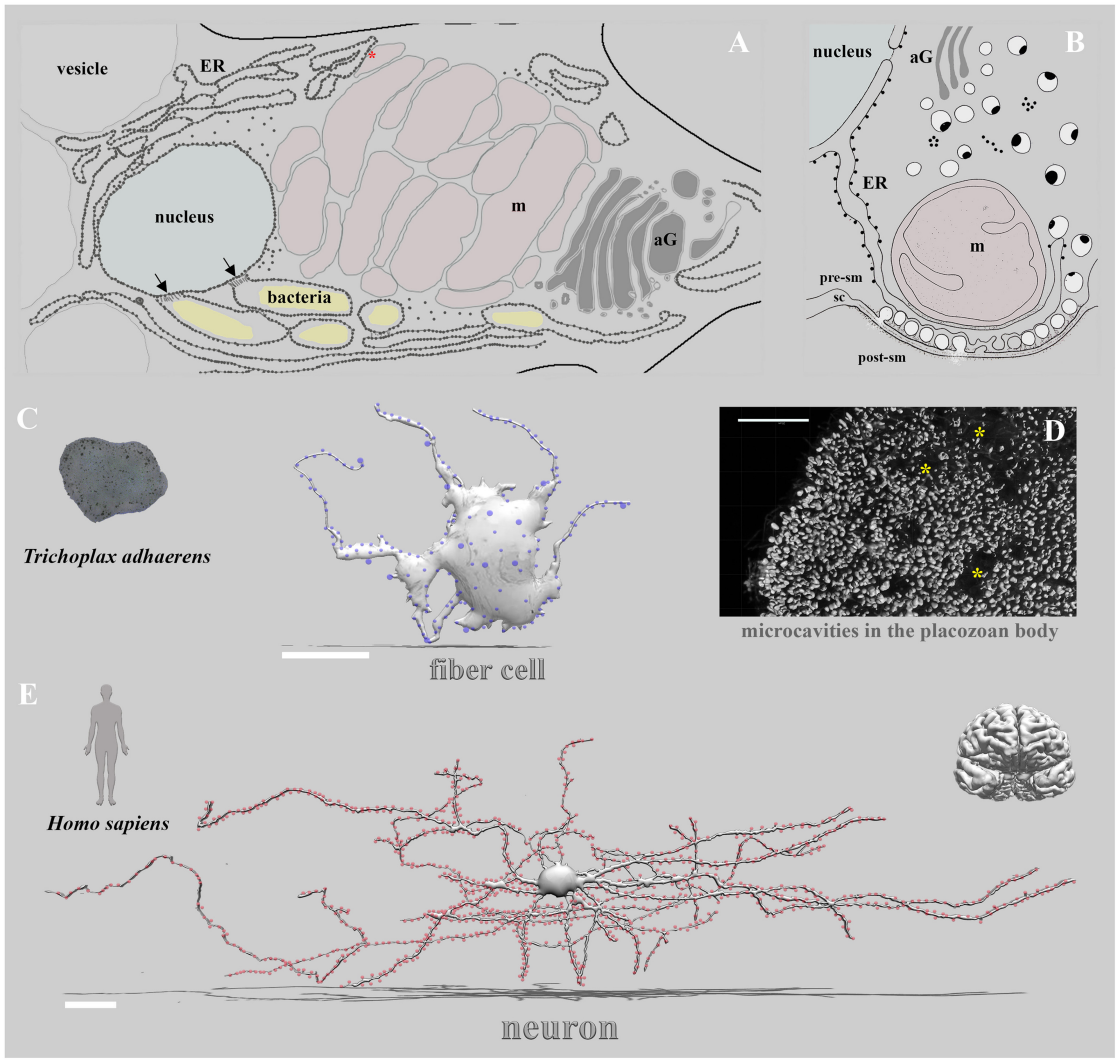
Non-synaptic vs. synaptic transmission: extreme cases of alternative integrative systems. Three remarkable examples of cells involved in the behavioral integrations are illustrated: the placozoan fiber cell **(A,C)**, unique ctenophore tripartite chemical synapse (**B**, modified from Hernandez-Nicaise, 1991, see text), and the pyramidal human neuron **(E)**. **(A)** Details of ultrastructural organization of the fiber cell in *Hoilungia hongkongensis* (modified from Romanova et al., 2021, Figure 5). Inside fiber cells, an extended endoplasmic reticulum entwined all cell compartments, especially mitochondria complex (formed presumed mitochondrial contact site—red asterisk), nucleus, and bacteria. Black arrows indicate specialized contact sites (Dumoux and Hayward, 2016) to the nucleus with encapsulated bacteria within ER-type structures with ribosomes (dotted areas). **(C)** The left image shows the organization disk-shaped nerveless placozoan, *Trichoplax*, which contains no recognized neurons, muscles, or sensory organs but displays coordinated behaviors and action potentials (Smith et al., 2015, 2019; Senatore et al., 2017; Armon et al., 2018; Varoqueaux et al., 2018; Fortunato and Aktipis, 2019; Romanova et al., 2020b). The middle image shows the schematic reconstruction of a placozoan fiber cell with prominent sites (blue dots) of putative secretory/paracrine (non-synaptic) regions (modified from scanning and transmission electrom microscopy datasets (Romanova et al., 2021). The right image **(D)** shows the microcavities (asterisks) (Romanova, 2019) as suggested regions of non-synaptic communications and integration in placozoans (Moroz et al., 2021b). **(E)** The reconstruction of a pyramidal neuron (modified from https://ai.googleblog.com/2021/06/a-browsable-petascale-reconstruction-of.html and Riessland et al., 2017; Shapson-Coe et al., 2021). Red dots are exemplar synapses on the pyramidal neuron. Blue dots are recognized vesicles and exosomes around the fiber cell **(C)**. Scale bars: Fiber cell—20μm; Neuron—50μm.

*Third*, many **transmitters are common cellular metabolites** or directly derived from cellular metabolites. In this capacity, signal molecules can also be food/energy sources (Moroz et al., 2021a), (e.g., amino acids such as glutamate and aspartate or small peptides) for specialized cells and symbionts or endoparasites. Thus, more compartmentalized [synapse-type] communication provided potential “protections” of signaling molecules from their consumption by other cells or symbionts/parasites. In the nerveless *Trichoplax* (Figure 1), the fiber cells seem to contribute to neuroid integrative, phagocytotic, immune, regenerative, and contractile functions (Grell and Ruthmann, 1991; Romanova et al., 2021). They also contain intracellular symbionts (Gruber-Vodicka et al., 2019; Kamm et al., 2019). But, in placozoans, highly localized signaling might occur without classical synapses.

There are also “*disadvantages*” of the highly localized synaptic transmission related to spatial limitations of integrative functions. Slow diffusion of signal molecules to other (more distant) targets could be “compensated” by the growth of neuroid processes, energetically very costly mechanisms. In complex, relatively large, and mobile animals, there was a parallel development of different systems for long-distance signalings, such as circulatory and immune systems. The predation and larger body sizes were essential factors (Monk and Paulin, 2014), triggering the origins and rapid evolution of the neural and synaptic organizations.

As a result, we might envision multiple trade-offs between speed, efficiency, and associated energy cost of non-synaptic volume transmission vs. highly localized synapses in the early evolution of animal communication systems. These two communication systems always co-exist in most extant animals. But how had chemical synapses evolved? There are three aspects to this question. (i) combinatorial selection of molecular components [modules], including recruitments of various adhesive molecules to bring presynaptic and postsynaptic complexes together, and/or cooption of gap junctions proteins for the same purposes (Ovsepian and Vesselkin, 2014; Ovsepian, 2017; Ovsepian et al., 2020); (ii) reorganization of intracellular and extracellular membrane domains in secretory cells and their targets to enhance signaling and communication efficiency; and (iii) preferential selection of chemically different transmitter classes for paracrine vs. synaptic communications. Below, we will briefly discuss these interconnected components of synaptic evolution.

### The Versatility of Secretory and Receptive Modules Is the Core of the Synaptic Origins

Natural selection might take advantage of several “preadaptations” to “build” synapses using a broad array of modular exocytosis machinery and adhesive molecules (cadherins, neurexins, neuroligins, immunoglobulins, complex receptive scaffolds from unicellular eukaryotes, etc.)—all these components were previously selected for other functions rather than to make synapses. It perfectly fits the definition of the ***exaptation*** as “characters evolved for other uses…, and later co-opted for their current role.” (Gould and Vrba, 1982). However, we must view a knot of such exaptations within contexts and constraints of each phyletic lineage of animals, their bodyplans, and their development. Equally important would be modeling the energetic cost of synapse formation, growth, and the maintenance of long neural processes (which consume a lot of energy to sustain their homeostasis, propagate electrical signals and secretory events). These factors, plus ecologies and behaviors of particular species, provide additional constraints to synaptic recruitments, synaptic architecture, and even preferential selection of “available” transmitters in one or another type of neural system.

Limited comparative, cell-specific molecular and physiological data from early-branching metazoan lineages prevent making final conclusions about the combinatorial logic and the scope of modularity within different synaptic architectures. However, we can state that most core molecular machinery, sufficient for synapse formation (secretory presynaptic and postsynaptic receptive modules), predated the origin of animals and their neural systems (Ryan and Grant, 2009; Moroz and Kohn, 2015, 2016; Ovsepian, 2017; Ovsepian et al., 2020). In unicellular and colonial eukaryotes, paracrine secretion is widely used for other functions, different from (neuro)transmitter signaling, such as digestion, phagocytosis, defense, immunity, and injury-regenerative responses, control of cell divisions and differentiation, etc.

## Endoplasmic Reticulum in Protoneuronal Secretory Cells Might Promote Synaptogenesis in Evolution

Over 60 years, scientists put forward the idea that neurons evolved from secretory cells (reviewed Moroz, 2014, 2021). But what kind of preadaptations in secretory cells (apart from exocytosis itself) might facilitate synaptogenesis? We hypothesize that the endoplasmic reticulum (ER) specialization toward enhanced secretion capabilities and associated dramatic increase of intercellular membranous structures could be essential factors that triggered and shaped early neuronal and synaptic evolution. Three outcomes of enhanced secretory functions relevant to synaptic evolutionary selection are summarized below.

### Increased Intracellular Membrane Space in Secretory Cells Drives Organelles' Interactions and Lipid Complexity

ER comprises more than half of the total cell membranes and occupies about 35% of cytoplasmic volume (Valm et al., 2017). However, the intracellular membranous space is more extensive in secretory cells and cells with extended neural processes such as axon-type terminals, which have elaborated ER even in distant neurites (Ozturk et al., 2020). Increased synthesis of peptides and other secretory molecules, their accumulation in ER and vesicles, and Ca^2+^- dependent vesicular release—all require a dramatic expansion of ER and its tightly coupled interactions with mitochondria, Golgi apparatus, and other organelles (Sassano and Agostinis, 2019). The expanded and highly heterogeneous lipid space (Harayama and Riezman, 2018; Santos and Preta, 2018) facilitates complex phase transitions among biomolecules, organelles (Bag et al., 2020; King et al., 2020), and synaptic vesicles (Rohrbough and Broadie, 2005). Lipid synthesis occurs in ER, and the current estimates suggest that more than 38,000 different identified lipids composed cellular membranes (Liebisch et al., 2020). The theory predicts ~180,000 lipid species distributed among eight major lipid categories (Brugger, 2014), supporting astonishing diversity of functions. Considering both enrichment of neural systems in lipids and the fact the ~75% of lipid diversity is found in the brain, we can say: ***neuronal evolution is the lipid revolution***. The molecular diversity of synapses (Rohrbough and Broadie, 2005) and neurons based on lipidomics can be even greater than described using scRNA-seq, and the plasma lipid composition is a neuron-/cell-type-specific feature (Neumann et al., 2019; Fitzner et al., 2020). The most imperative are the data illustrating that the differences in lipid composition between cell-type plasma membranes are smaller than differences between organellar membranes in a given cell (Symons et al., 2021). In other words, each organelle might have its unique lipidome (Symons et al., 2021), and cell-to-cell communications by extracellular vesicles is a little-explored route of lipid signaling (Barber and Raben, 2019; Skotland et al., 2020).

### Different Fates of Lipophilic vs. Lipophobic Transmitters

Increased lipid diversity of plasma and intracellular membranes shaped evolutionary recruitments of different transmitters in synaptic architectures. Small transmitters directly interact with membranes and can be broadly divided into two groups. Lipophilic transmitters (i.e., melatonin, serotonin, histamine, dopamine, noradrenaline, adrenaline, and adenosine) had high lipid partition coefficients (Wang et al., 2011; Postila et al., 2016; Engberg et al., 2020; Josey et al., 2020; Parkkila and Viitala, 2020) and can operate in receptor-independent mechanisms by changing lipid dynamics (Dey et al., 2021). They accumulated within surface membrane layers (e.g., postsynaptic membranes) with enhanced planar 2D diffusions. In other words, they act according to the 3D → 2D diffusion scheme (Postila and Rog, 2020). In contrast, glutamate, aspartate, glycine, and GABA are primarily lipophobic molecules. Accordingly, they work as classical transmitters in 3D diffusion space, but this situation can be changed in the presence of Ca^2+^ (Perez-Isidoro and Ruiz-Suarez, 2016). Acetylcholine has an intermediate position in its interactions with lipids (Postila et al., 2016; Postila and Rog, 2020).

Consequently, there are selection constraints in the synaptic receptor architecture designs (Postila et al., 2016; Postila and Rog, 2020). In many G-protein coupled receptors for lipophilic transmitters, the ligand-binding sites are predominantly (but not exclusively) hidden in the membrane (Postila and Rog, 2020). In contrast, the binding sites are primarily in the extracellular space for lipophobic transmitter receptors (e.g., iGluRs, mGluRs or nicotinic receptors). These physical properties of signal molecules might contribute to the fact that the first synapses utilized lipophobic transmitters (Glu, Asp, GABA), in addition to neuropeptides, as in extant ctenophores and cnidarians (Moroz et al., 2021a,b). The recruitments of lipophilic transmitters in neural systems and synapses seemingly occurred later in evolution, only in bilaterians (Moroz et al., 2021b). Furthermore, glutamate and acetylcholine were recruited primarily as fast excitatory neurotransmitters in the neocortex and neuromuscular junctions of vertebrates, respectively. The reverse situation occurred in insects, where glutamate was recruited as a fast neuromuscular transmitter (Jan and Jan, 1976). More likely, it is a reflection of evolutionary recruitment games under similar physical and chemical constraints for fast transmission in different evolutionary lineages. Together with their chemical stability and fast uptake/inactivation and coupling to bioenergetic, the lipophobic properties of transmitters might provide selective advantages for rapid synaptic communication dynamics.

### Integrative Functions of ER as a Hub of Neuronal/Synaptic Innovations

ER physically and chemically interacts with cellular organelles using vesicular and non-vesicular lipid transport (Holthuis and Menon, 2014) at specialized membrane contact sites (MCS, Levine, 2004; Phillips and Voeltz, 2016; Ruiz-Lopez et al., 2021). MCS support complex interactions with mitochondria (Schlattner et al., 2014; Kannan et al., 2017; Wong et al., 2019) via tethered regions of ER known as **m****itochondria-****a****ssociated** **m****embranes** or MAM (Allen et al., 1989; Helle et al., 2013).

Specialized lipid chaperons can control Ca-dependent regulations and dynamic composition of lipid rafts at MCS [e.g., Sigma 1 receptor (Zhemkov et al., 2021a)], also acting as hubs of inter-organelle communications and signaling (Zhemkov et al., 2021c). Not surprisingly that such regulations at MCS and mitochondria do control neuropeptide asymmetric distribution and secretion (Valadas et al., 2018; Zhao et al., 2018), cell death (Prudent et al., 2015) and contribute to mechanisms underlying neurological disorders (Schon and Area-Gomez, 2013; Zhemkov et al., 2021b) and synaptopathies (Di Miceli et al., 2020).

ER is major calcium storage and the system for Ca^2+^ homeostasis (Berridge, 1998). A continuous, highly extended ER network is viewed as an “intracellular highway” or a much faster route for Ca^2+^ tunneling over long distances due to little Ca^2+^ buffering in the ER lumen in secretory cells (Petersen et al., 2017) and, perhaps, in neurons too.

In other words, ER has been termed a “***neuron within a neuron*”** (Berridge, 1998, 2002). Boosted ER-Ca^2+^/MCS/MAM systems in early secretory cells [or protoneurons] were ideally suited for developing a polarized release of all classes of transmitters with versatile preadaptations for divergent synaptic evolution in later Precambrian animals.

MAMs could also be viewed as an ancestral prototype of intracellular communication at the synapse. **Cellular stress and immunity responses** form dynamic tethering of ***signaling*** ***synapses between ER and MAM*** (Horner et al., 2011). MAM could be further co-evolved to support cell-cell synaptic interactions with high bioenergetic “demands” by coupling the same components [mitochondria (energy) and ER (secretion)] in presynaptic (and even postsynaptic) membranes.

The tripartite synapses in ctenophores (Figure 1B), with layered arrangements of secretory vesicles, ER, and mitochondrion (Hernandez-Nicaise, 1991), are the perfect examples of the membranous structural organization in one of the earliest synapse designs (Moroz, 2015). These asymmetrical synapses contained three distinct layers of organelles, forming a so-called “presynaptic triad” (Hernandez-Nicaise, 1973, 1974, 1991): (i) a single layer of synaptic vesicles lining the presynaptic membrane, (ii) a cistern of agranular endoplasmic reticulum just above the row of vesicles, followed by (iii) one or several mitochondria with presumed MAM type contacts. The postsynaptic density and active zones, however, are less prominent in ctenophore synapses. ER-mitochondria relationships can also be noted in some cnidarian synapses (Anderson, 1985; Anderson and Grunert, 1988; Anderson and Spencer, 1989).

Mitochondria complexes and elaborated ER structures are characteristics of the fiber cells of placozoans (Figures 1A,C). These tetraploid cells can coordinate several interrelated functions such as systemic feeding with bacterial phagocytosis and immunity responses. As a result, we view a meshwork of fiber cells [and associated small star-like cells (Romanova et al., 2021)] as an organism-scale integrative or homeostatic system, potentially involved in systemic injury and regeneration responses, perhaps even in morphogenesis. This type of system can be close to the hypothetical protoneuronal organization, which initially evolved to control morphogenesis in first nerveless metazoans (Fields et al., 2020).

## Questions and Perspectives

We only scratched the surface of the problem of synaptic selection. Early interdependence and ancestral relationships of innate immune and neural systems is another poorly investigated layer in the evolution of intercellular communications. The landscape for developing immune functions is similar to neural control due to many shared secretory products, lipid and ER rearrangements and conceptually shared features of neuronal and immune synapses with similar adhesive molecules. In due course, both systems (co-)evolved as responses to stress/injury factors and recognition of self vs. foreign RNA, DNA, protein, and cell invasions. Remarkably, ER-MAMs signaling also plays an essential role in innate immunity against RNA virus infection: as a platform for inducing an immune response and regulating viral replication. MAM tethering ER to mitochondria and peroxisome(s) form immune synapses during RNA virus invasion (Horner et al., 2011, 2015). Thus, it would be intriguing to think that some architectures of neural systems evolved as a branch of immune communications. Injury-induced regeneration signaling can be a universal exaptation for immune and neural systems, both adopted for faster responses. The growing diversity and compartmentalization of lipids and ER further promoted cell plasticity, forming more localized immune and neural synapses, often recruiting the same transmitters (e.g., histamine and serotonin, glutamate, and GABA), as well as multiplicity of small signaling peptides. Ultimately, as all things in nature, membrane-membrane and cell-cell communications should be physically closer to be efficient.

## Author Contributions

DR: visualization. All authors contributed to the article and approved the submitted version.

## Author Disclaimer

The content is solely the responsibility of the authors and does not necessarily represent the official views of the National Institutes of Health.

## Conflict of Interest

The authors declare that the research was conducted in the absence of any commercial or financial relationships that could be construed as a potential conflict of interest.

## Publisher's Note

All claims expressed in this article are solely those of the authors and do not necessarily represent those of their affiliated organizations, or those of the publisher, the editors and the reviewers. Any product that may be evaluated in this article, or claim that may be made by its manufacturer, is not guaranteed or endorsed by the publisher.

## References

[B1] Allen R. D. Schroeder C. C. Fok A. K. (1989). An investigation of mitochondrial inner membranes by rapid-freeze deep-etch techniques. J. Cell. Biol. 108, 2233–2240. 10.1083/jcb.108.6.2233 2525561 PMC2115613

[B2] Anderson P. A. (1985). Physiology of a bidirectional, excitatory, chemical synapse. J. Neurophysiol. 53, 821–835. 10.1152/jn.1985.53.3.821 2984356

[B3] Anderson P. A. Grunert U. (1988). Three-dimensional structure of bidirectional, excitatory chemical synapses in the jellyfish *Cyanea capillata*. Synapse 2, 606–613. 10.1002/syn.890020605 2905537

[B4] Anderson P. A. Spencer A. N. (1989). The importance of cnidarian synapses for neurobiology. J. Neurobiol. 20, 435–457. 10.1002/neu.480200513 2568389

[B5] Armon S. Bull M. S. Aranda-Diaz A. Prakash M. (2018). Ultrafast epithelial contractions provide insights into contraction speed limits and tissue integrity. Proc. Natl. Acad. Sci. U. S. A. 115, E10333–E10341. 10.1073/pnas.1802934115 30309963 PMC6217427

[B6] Bag N. Ramezani M. Holowka D. A. Baird B. A. (2020). Bringing light to ER contacts and a new phase in organelle communication. Proc. Natl. Acad. Sci. U. S. A. 117, 9668–9670. 10.1073/pnas.2003620117 32345722 PMC7211920

[B7] Barber C. N. Raben D. M. (2019). Lipid metabolism crosstalk in the brain: glia and neurons. Front. Cell. Neurosci. 13:212. 10.3389/fncel.2019.00212 31164804 PMC6536584

[B8] Berridge M. J. (1998). Neuronal calcium signaling. Neuron 21, 13–26. 10.1016/S0896-6273(00)80510-3 9697848

[B9] Berridge M. J. (2002). The endoplasmic reticulum: a multifunctional signaling organelle. Cell. Calcium 32, 235–249. 10.1016/S0143416002001823 12543086

[B10] Brugger B. (2014). Lipidomics: analysis of the lipid composition of cells and subcellular organelles by electrospray ionization mass spectrometry. Annu. Rev. Biochem. 83, 79–98. 10.1146/annurev-biochem-060713-035324 24606142

[B11] Burbulla L. F. Song P. Mazzulli J. R. Zampese E. Wong Y. C. Jeon S.. (2017). Dopamine oxidation mediates mitochondrial and lysosomal dysfunction in Parkinson's disease. Science 357, 1255–1261. 10.1126/science.aam9080 28882997 PMC6021018

[B12] Dey S. Surendran D. Engberg O. Gupta A. Fanibunda S. E. Das A.. (2021). Altered membrane mechanics provides a receptor-independent pathway for serotonin action. Chemistry 27, 7533–7541. 10.1002/chem.202100328 33502812 PMC8252079

[B13] Di Miceli M. Bosch-Bouju C. Laye S. (2020). PUFA and their derivatives in neurotransmission and synapses: a new hallmark of synaptopathies. Proc. Nutr. Soc. 1–16. 10.1017/S0029665120000129 32299516

[B14] Dumoux M. Hayward R. D. (2016). Membrane contact sites between pathogen-containing compartments and host organelles. Biochim. Biophys. Acta 1861, 895–899. 10.1016/j.bbalip.2016.01.018 26825687

[B15] Engberg O. Bochicchio A. Brandner A. F. Gupta A. Dey S. Bockmann R. A.. (2020). Serotonin alters the phase equilibrium of a ternary mixture of phospholipids and cholesterol. Front. Physiol. 11:578868. 10.3389/fphys.2020.578868 33192582 PMC7645218

[B16] Fields C. Bischof J. Levin M. (2020). Morphological coordination: a common ancestral function unifying neural and non-neural signaling. Physiology 35, 16–30. 10.1152/physiol.00027.2019 31799909

[B17] Fitzner D. Bader J. M. Penkert H. Bergner C. G. Su M. Weil M. T.. (2020). Cell-type- and brain-region-resolved mouse brain lipidome. Cell. Rep. 32:108132. 10.1016/j.celrep.2020.108132 32937123

[B18] Fortunato A. Aktipis A. (2019). Social feeding behavior of *Trichoplax adhaerens*. Front. Ecol. Evol. 7:19. 10.3389/fevo.2019.00019 31667165 PMC6821444

[B19] Gould S. J. Vrba E. S. (1982). Exaptation—a missing term in the science of form. Paleobiology 8, 4–15. 10.1017/S0094837300004310

[B20] Grell K. G. Ruthmann A. (1991). Placozoa, in Microscopic Anatomy of Invertebrates, ed Harrison F. W. (New York, NY: Wiley-Liss), 13–27.

[B21] Gruber-Vodicka H. R. Leisch N. Kleiner M. Hinzke T. Liebeke M. Mcfall-Ngai M.. (2019). Two intracellular and cell type-specific bacterial symbionts in the placozoan Trichoplax H2. Nat. Microbiol. 4, 1465–1474. 10.1038/s41564-019-0475-9 31182796 PMC6784892

[B22] Halanych K. M. Whelan N. V. Kocot K. M. Kohn A. B. Moroz L. L. (2016). Miscues misplace sponges. Proc. Natl. Acad. Sci. U. S. A. 113, E946–947. 10.1073/pnas.1525332113 26862177 PMC4776479

[B23] Harayama T. Riezman H. (2018). Understanding the diversity of membrane lipid composition. Nat. Rev. Mol. Cell. Biol. 19, 281–296. 10.1038/nrm.2017.138 29410529

[B24] Helle S. C. Kanfer G. Kolar K. Lang A. Michel A. H. Kornmann B. (2013). Organization and function of membrane contact sites. Biochim. Biophys. Acta 1833, 2526–2541. 10.1016/j.bbamcr.2013.01.028 23380708

[B25] Hernandez-Nicaise M.-L. (1991). Ctenophora, in Microscopic Anatomy of Invertebrates: Placozoa, Porifera, Cnidaria, and Ctenophora, eds Harrison F. W. Westfall J. A. (New York, NY: Wiley), 359–418.

[B26] Hernandez-Nicaise M. L. (1973). The nervous system of ctenophores. III. Ultrastructure of synapses. J. Neurocytol. 2, 249–263. 10.1007/BF01104029 9224490

[B27] Hernandez-Nicaise M. L. (1974). Ultrastructural evidence for a sensory-motor neuron in Ctenophora. Tissue Cell 6, 43–47. 10.1016/0040-8166(74)90021-4 4151616

[B28] Holthuis J. C. Menon A. K. (2014). Lipid landscapes and pipelines in membrane homeostasis. Nature 510, 48–57. 10.1038/nature13474 24899304

[B29] Horner S. M. Liu H. M. Park H. S. Briley J. Gale M. Jr. (2011). Mitochondrial-associated endoplasmic reticulum membranes (MAM) form innate immune synapses and are targeted by hepatitis C virus. Proc. Natl. Acad. Sci. U. S. A. 108, 14590–14595. 10.1073/pnas.1110133108 21844353 PMC3167523

[B30] Horner S. M. Wilkins C. Badil S. Iskarpatyoti J. Gale M Jr. (2015). Proteomic analysis of mitochondrial-associated ER membranes (MAM) during RNA virus infection reveals dynamic changes in protein and organelle trafficking. PLoS ONE 10:e0117963. 10.1371/journal.pone.0117963 25734423 PMC4348417

[B31] Jan L. Y. Jan Y. N. (1976). L-glutamate as an excitatory transmitter at the *Drosophila* larval neuromuscular junction. J. Physiol. 262, 215–236. 10.1113/jphysiol.1976.sp011593 186587 PMC1307638

[B32] Josey B. P. Heinrich F. Silin V. Losche M. (2020). Association of model neurotransmitters with lipid bilayer membranes. Biophys. J. 118, 1044–1057. 10.1016/j.bpj.2020.01.016 32032504 PMC7063487

[B33] Kamm K. Osigus H. J. Stadler P. F. Desalle R. Schierwater B. (2019). Genome analyses of a placozoan rickettsial endosymbiont show a combination of mutualistic and parasitic traits. Sci. Rep. 9:17561. 10.1038/s41598-019-54037-w 31772223 PMC6879607

[B34] Kannan M. Lahiri S. Liu L. K. Choudhary V. Prinz W. A. (2017). Phosphatidylserine synthesis at membrane contact sites promotes its transport out of the ER. J. Lipid Res. 58, 553–562. 10.1194/jlr.M072959 28119445 PMC5335585

[B35] Kapli P. Telford M. J. (2020). Topology-dependent asymmetry in systematic errors affects phylogenetic placement of Ctenophora and Xenacoelomorpha. Sci. Adv. 6, 1–11. 10.1126/sciadv.abc5162 33310849 PMC7732190

[B36] King C. Sengupta P. Seo A. Y. Lippincott-Schwartz J. (2020). ER membranes exhibit phase behavior at sites of organelle contact. Proc. Natl. Acad. Sci. U. S. A. 117, 7225–7235. 10.1073/pnas.1910854117 32179693 PMC7132286

[B37] Levine T. (2004). Short-range intracellular trafficking of small molecules across endoplasmic reticulum junctions. Trends Cell. Biol. 14, 483–490. 10.1016/j.tcb.2004.07.017 15350976

[B38] Liebisch G. Fahy E. Aoki J. Dennis E. A. Durand T. Ejsing C. S.. (2020). Update on LIPID MAPS classification, nomenclature, and shorthand notation for MS-derived lipid structures. J. Lipid Res. 61, 1539–1555. 10.1194/jlr.S120001025 33037133 PMC7707175

[B39] Mackie G. O. (1970). Neuroid conduction and the evolution of conducting tissues. Q. Rev. Biol. 45, 319–332. 10.1086/406645 4395914

[B40] Mackie G. O. (1990). The elementary nervous sytems revisited. Am. Zool. 30, 907–920. 10.1093/icb/30.4.907

[B41] Miguel-Tomé S. Llinás R. R. (2021). Broadening the definition of a nervous system to better understand the evolution of plants and animals. Plant Signal. Behav. 2, 1–18. 10.1080/15592324.2021.1927562 34120565 PMC8331040

[B42] Monk T. Paulin M. G. (2014). Predation and the origin of neurones. Brain Behav. Evol. 84, 246–261. 10.1159/000368177 25472692

[B43] Moroz L. L. (2009). On the independent origins of complex brains and neurons. Brain Behav. Evol. 74, 177–190. 10.1159/000258665 20029182 PMC2855278

[B44] Moroz L. L. (2014). The genealogy of genealogy of neurons. Commun. Integr. Biol. 7:e993269. 10.4161/19420889.2014.993269 26478767 PMC4594457

[B45] Moroz L. L. (2015). Convergent evolution of neural systems in ctenophores. J. Exp. Biol. 218, 598–611. 10.1242/jeb.110692 25696823 PMC4334147

[B46] Moroz L. L. (2021). Multiple origins of neurons from secretory cells. Front. Cell Dev. Biol. 9:1–9. 10.3389/fcell.2021.669087 34307354 PMC8293673

[B47] Moroz L. L. Kocot K. M. Citarella M. R. Dosung S. Norekian T. P. Povolotskaya I. S.. (2014). The ctenophore genome and the evolutionary origins of neural systems. Nature 510, 109–114. 10.1038/nature13400 24847885 PMC4337882

[B48] Moroz L. L. Kohn A. B. (2015). Unbiased view of synaptic and neuronal gene complement in ctenophores: are there pan-neuronal and pan-synaptic genes across metazoa? Integr. Comp. Biol. 55, 1028–1049. 10.1093/icb/icv104 26454853 PMC4836450

[B49] Moroz L. L. Kohn A. B. (2016). Independent origins of neurons and synapses: insights from ctenophores. Philos. Trans. R. Soc. Lond. B. Biol. Sci. 371:20150041. 10.1098/rstb.2015.0041 26598724 PMC4685580

[B50] Moroz L. L. Nikitin M. A. Poličar P. G. Kohn A. B. Romanova D. Y. (2021a). Evolution of glutamatergic signaling and synapses. Neuropharmacology. 10.1016/j.neuropharm.2021.108740. [Epub ahead of print]. 34343611 PMC9233959

[B51] Moroz L. L. Romanova D. Y. Kohn A. B. (2021b). Neural versus alternative integrative systems: molecular insights into origins of neurotransmitters. Philos. Trans. R. Soc. Lond. B Biol. Sci. 376:20190762. 10.1098/rstb.2019.0762 33550949 PMC7935107

[B52] Moroz L. L. Romanova D. Y. Nikitin M. A. Sohn D. Kohn A. B. Neveu E.. (2020a). The diversification and lineage-specific expansion of nitric oxide signaling in Placozoa: insights in the evolution of gaseous transmission. Sci. Rep. 10:13020. 10.1038/s41598-020-69851-w 32747709 PMC7400543

[B53] Moroz L. L. Sohn D. Romanova D. Y. Kohn A. B. (2020b). Microchemical identification of enantiomers in early-branching animals: lineage-specific diversification in the usage of D-glutamate and D-aspartate. Biochem. Biophys. Res. Commun. 527, 947–952. 10.1016/j.bbrc.2020.04.135 32439167

[B54] Neumann E. K. Ellis J. F. Triplett A. E. Rubakhin S. S. Sweedler J. V. (2019). Lipid analysis of 30000 individual rodent cerebellar cells using high-resolution mass spectrometry. Anal. Chem. 91, 7871–7878. 10.1021/acs.analchem.9b01689 31122012 PMC6660023

[B55] Nikitin M. (2015). Bioinformatic prediction of *Trichoplax adhaerens* regulatory peptides. Gen. Comp. Endocrinol. 212, 145–155. 10.1016/j.ygcen.2014.03.049 24747483

[B56] Ovsepian S. V. (2017). The birth of the synapse. Brain Struct. Funct. 222, 3369–3374. 10.1007/s00429-017-1459-2 28612096

[B57] Ovsepian S. V. O'leary V. B. Vesselkin N. P. (2020). Evolutionary origins of chemical synapses. Vitam. Horm. 114, 1–21. 10.1016/bs.vh.2020.04.009 32723540

[B58] Ovsepian S. V. Vesselkin N. P. (2014). Wiring prior to firing: the evolutionary rise of electrical and chemical modes of synaptic transmission. Rev. Neurosci. 25, 821–832. 10.1515/revneuro-2014-0037 25051277

[B59] Ozturk Z. O'kane C. J. Perez-Moreno J. J. (2020). Axonal endoplasmic reticulum dynamics and its roles in neurodegeneration. Front. Neurosci. 14:48. 10.3389/fnins.2020.00048 32116502 PMC7025499

[B60] Parkkila P. Viitala T. (2020). Partitioning of catechol derivatives in lipid membranes: implications for substrate specificity to catechol-O-methyltransferase. ACS Chem. Neurosci. 11, 969–978. 10.1021/acschemneuro.0c00049 32101397 PMC7145343

[B61] Perez-Isidoro R. Ruiz-Suarez J. C. (2016). Calcium and protons affect the interaction of neurotransmitters and anesthetics with anionic lipid membranes. Biochim. Biophys. Acta 1858, 2215–2222. 10.1016/j.bbamem.2016.06.017 27362370

[B62] Petersen O. H. Courjaret R. Machaca K. (2017). Ca(2+) tunnelling through the ER lumen as a mechanism for delivering Ca(2+) entering via store-operated Ca(2+) channels to specific target sites. J. Physiol. 595, 2999–3014. 10.1113/JP272772 28181236 PMC5430212

[B63] Phillips M. J. Voeltz G. K. (2016). Structure and function of ER membrane contact sites with other organelles. Nat. Rev. Mol. Cell. Biol. 17, 69–82. 10.1038/nrm.2015.8 26627931 PMC5117888

[B64] Postila P. A. Rog T. (2020). A perspective: active role of lipids in neurotransmitter dynamics. Mol. Neurobiol. 57, 910–925. 10.1007/s12035-019-01775-7 31595461 PMC7031182

[B65] Postila P. A. Vattulainen I. Rog T. (2016). Selective effect of cell membrane on synaptic neurotransmission. Sci. Rep. 6:19345. 10.1038/srep19345 26782980 PMC4725992

[B66] Prudent J. Zunino R. Sugiura A. Mattie S. Shore G. C. Mcbride H. M. (2015). MAPL SUMOylation of Drp1 stabilizes an ER/mitochondrial platform required for cell death. Mol. Cell 59, 941–955. 10.1016/j.molcel.2015.08.001 26384664

[B67] Redmond A. K. McLysaght A. (2021). Evidence for sponges as sister to all other animals from partitioned phylogenomics with mixture models and recoding. Nat. Commun. 12:1783. 10.1038/s41467-021-22074-7 33741994 PMC7979703

[B68] Riessland M. Kolisnyk B. Greengard P. (2017). Reactive dopamine leads to triple trouble in nigral neurons. Biochemistry 56, 6409–6410. 10.1021/acs.biochem.7b01057 29188990

[B69] Rohrbough J. Broadie K. (2005). Lipid regulation of the synaptic vesicle cycle. Nat. Rev. Neurosci. 6, 139–150. 10.1038/nrn1608 15685219

[B70] Romanova D. Y. (2019). Cell types diversity of H4 haplotype Placozoa sp. Mar. Biol. J. 4, 81–90. 10.21072/mbj.2019.04.1.07

[B71] Romanova D. Y. Heyland A. Sohn D. Kohn A. B. Fasshauer D. Varoqueaux F.. (2020a). Glycine as a signaling molecule and chemoattractant in *Trichoplax* (Placozoa): insights into the early evolution of neurotransmitters. Neuroreport 31, 490–497. 10.1097/WNR.0000000000001436 32243353

[B72] Romanova D. Y. Smirnov I. V. Nikitin M. A. Kohn A. B. Borman A. I. Malyshev A. Y.. (2020b). Sodium action potentials in placozoa: insights into behavioral integration and evolution of nerveless animals. Biochem. Biophys. Res. Commun. 532, 120–126. 10.1016/j.bbrc.2020.08.020 32828537 PMC8214824

[B73] Romanova D. Y. Varoqueaux F. Daraspe J. Nikitin M. A. Eitel M. Fasshauer D.. (2021). Hidden cell diversity in Placozoa: ultrastructural insights from *Hoilungia hongkongensis*. Cell Tissue Res. 1–15. 10.1007/s00441-021-03459-y 33876313 PMC8523601

[B74] Ruiz-Lopez N. Perez-Sancho J. Esteban Del Valle A. Haslam R. P. Vanneste S. Catala R.. (2021). Synaptotagmins at the endoplasmic reticulum-plasma membrane contact sites maintain diacylglycerol homeostasis during abiotic stress. Plant Cell. 1–23. 10.1093/plcell/koab122. [Epub ahead of print]. 33944955 PMC8364230

[B75] Ryan T. J. Grant S. G. (2009). The origin and evolution of synapses. Nat. Rev. Neurosci. 10, 701–712. 10.1038/nrn2717 19738623

[B76] Santos A. L. Preta G. (2018). Lipids in the cell: organisation regulates function. Cell. Mol. Life Sci. 75, 1909–1927. 10.1007/s00018-018-2765-4 29427074 PMC11105414

[B77] Sassano M. L. Agostinis P. (2019). Staying in touch: taking a closer look at ER-Golgi contact sites. J. Cell Biol. 218, 729–731. 10.1083/jcb.201901039 30733234 PMC6400561

[B78] Schlattner U. Tokarska-Schlattner M. Rousseau D. Boissan M. Mannella C. Epand R.. (2014). Mitochondrial cardiolipin/phospholipid trafficking: the role of membrane contact site complexes and lipid transfer proteins. Chem. Phys. Lipids 179, 32–41. 10.1016/j.chemphyslip.2013.12.008 24373850

[B79] Schon E. A. Area-Gomez E. (2013). Mitochondria-associated ER membranes in Alzheimer disease. Mol. Cell. Neurosci. 55, 26–36. 10.1016/j.mcn.2012.07.011 22922446

[B80] Senatore A. Reese T. S. Smith C. L. (2017). Neuropeptidergic integration of behavior in *Trichoplax adhaerens*, an animal without synapses. J. Exp. Biol. 220, 3381–3390. 10.1242/jeb.162396 28931721 PMC5612019

[B81] Shapson-Coe A. Januszewski M. Berger D. R. Pope A. Wu Y. Blakely T.. (2021). A connectomic study of a petascale fragment of human cerebral cortex. BioRxiv [Preprint]. 10.1101/2021.05.29.446289

[B82] Skotland T. Sagini K. Sandvig K. Llorente A. (2020). An emerging focus on lipids in extracellular vesicles. Adv. Drug Deliv. Rev. 159, 308–321. 10.1016/j.addr.2020.03.002 32151658

[B83] Smith C. L. Pivovarova N. Reese T. S. (2015). Coordinated feeding behavior in *Trichoplax*, an animal without Synapses. PLoS ONE 10:e0136098. 10.1371/journal.pone.0136098 26333190 PMC4558020

[B84] Smith C. L. Reese T. S. Govezensky T. Barrio R. A. (2019). Coherent directed movement toward food modeled in *Trichoplax*, a ciliated animal lacking a nervous system. Proc. Natl. Acad. Sci. U. S. A. 116, 8901–8908. 10.1073/pnas.1815655116 30979806 PMC6500112

[B85] Symons J. L. Cho K. J. Chang J. T. Du G. Waxham M. N. Hancock J. F.. (2021). Lipidomic atlas of mammalian cell membranes reveals hierarchical variation induced by culture conditions, subcellular membranes, and cell lineages. Soft Matter 17, 288–297. 10.1039/D0SM00404A 32451522 PMC7688498

[B86] Telford M. J. Moroz L. L. Halanych K. M. (2016). Evolution: a sisterly dispute. Nature 529, 286–287. 10.1038/529286a 26791714

[B87] Valadas J. S. Esposito G. Vandekerkhove D. Miskiewicz K. Deaulmerie L. Raitano S.. (2018). ER lipid defects in neuropeptidergic neurons impair sleep patterns in Parkinson's disease. Neuron 98, 1155–1169 e1156. 10.1016/j.neuron.2018.05.022 29887339

[B88] Valm A. M. Cohen S. Legant W. R. Melunis J. Hershberg U. Wait E.. (2017). Applying systems-level spectral imaging and analysis to reveal the organelle interactome. Nature 546, 162–167. 10.1038/nature22369 28538724 PMC5536967

[B89] Varoqueaux F. Williams E. A. Grandemange S. Truscello L. Kamm K. Schierwater B.. (2018). High cell diversity and complex peptidergic signaling underlie placozoan behavior. Curr. Biol. 28, 3495–3501 e3492. 10.1016/j.cub.2018.08.067 30344118

[B90] Wang C. Ye F. Velardez G. F. Peters G. H. Westh P. (2011). Affinity of four polar neurotransmitters for lipid bilayer membranes. J. Phys. Chem. B 115, 196–203. 10.1021/jp108368w 21158460

[B91] Whelan N. V. Kocot K. M. Moroz L. L. Halanych K. M. (2015). Error, signal, and the placement of Ctenophora sister to all other animals. Proc. Natl. Acad. Sci. U. S. A. 112, 5773–5778. 10.1073/pnas.1503453112 25902535 PMC4426464

[B92] Whelan N. V. Kocot K. M. Moroz T. P. Mukherjee K. Williams P. Paulay G.. (2017). Ctenophore relationships and their placement as the sister group to all other animals. Nat. Ecol. Evol. 1, 1737–1746. 10.1038/s41559-017-0331-3 28993654 PMC5664179

[B93] Wong Y. C. Kim S. Peng W. Krainc D. (2019). Regulation and function of mitochondria-lysosome membrane contact sites in cellular homeostasis. Trends Cell. Biol. 29, 500–513. 10.1016/j.tcb.2019.02.004 30898429 PMC8475646

[B94] Zhao T. Hao Y. Kaplan J. M. (2018). Axonal mitochondria modulate neuropeptide secretion through the hypoxic stress response in *Caenorhabditis elegans*. Genetics 210, 275–285. 10.1534/genetics.118.301014 30049781 PMC6116974

[B95] Zhemkov V. Ditlev J. A. Lee W. R. Wilson M. Liou J. Rosen M. K.. (2021a). The role of sigma 1 receptor in organization of endoplasmic reticulum signaling microdomains. Elife 10:e65192. 10.7554/eLife.65192 33973848 PMC8112866

[B96] Zhemkov V. Geva M. Hayden M. R. Bezprozvanny I. (2021b). Sigma-1 receptor (S1R) interaction with cholesterol: mechanisms of S1R activation and its role in neurodegenerative diseases. Int. J. Mol. Sci. 22:4082. 10.3390/ijms22084082 33920913 PMC8071319

[B97] Zhemkov V. Liou J. Bezprozvanny I. (2021c). Sigma 1 receptor, cholesterol and endoplasmic reticulum contact sites. Contact 4, 1–7. 10.1177/25152564211026505 PMC10243589 37366370

